# Impact of Glutathione-S-Transferases (GST) Polymorphisms and Hypermethylation of Relevant Genes on Risk of Prostate Cancer Biochemical Recurrence: A Meta-Analysis

**DOI:** 10.1371/journal.pone.0074775

**Published:** 2013-09-23

**Authors:** Rui Chen, Shancheng Ren, Tong Meng, Josephine Aguilar, Yinghao Sun

**Affiliations:** 1 Department of Urology, Shanghai Changhai Hospital, Second Military Medical University, Shanghai, China; 2 Department of Orthopedics, Shanghai Changzheng Hospital, Second Military Medical University, Shanghai, China; 3 Department of Pathology and Laboratory Medicine, David Geffen School of Medicine at University of California, Los Angeles, Los Angeles, California, United States of America; The University of Texas M. D. Anderson Cancer Center, United States of America

## Abstract

**Introduction:**

Accurate prediction of the biochemical recurrence (BCR) is critical for patients after intended curative therapy like radical prostatectomy (RP) or definitive radiotherapy for prostate cancer. Glutathione-S-transferases polymorphisms as well as hypermethylation of GSTP1 and functional genes in carcinogenesis, including tumor suppression gene (APC), hormone receptor that regulates cell growth and differentiation gene (RARbeta) were reported to be associated with BCR. Nevertheless, the reported results are inconsistent. To evaluate the relationship between glutathione-S-transferases polymorphisms and hypermethylation of these genes and the risk of prostate cancer BCR, we carried out a meta-analysis of the published studies.

**Methods and Materials:**

We performed a search in Medline, Embase and CNKI database with GST, APC, RARbeta in combination with single nucleotide polymorphism, hypermethylation, prostate cancer and recurrence. Languages were restricted to English and Chinese.

**Results:**

Our study included 4 case-control studies and 7 cohort studies including 12 data sets and 3,037 prostate cancer patients. We confirmed that APC hypermethylation is associated with a modest hazard for biochemical recurrence after RP (HR = 1.85, 95%CI = 1.12–3.06). We also suggest GSTP1 polymorphism and CpG hypermethylation tested in serum are associated with BCR (HR = 1.94, 95%CI = 1.13–3.34). We also identified a possible association between GSTM1 null polymorphism and prostate cancer biochemical recurrence risk with borderline significance (HR = 1.29, 95%CI = 0.97–1.71).

**Conclusion:**

To our knowledge, this is the first meta-analysis evaluating the relationship of polymorphisms and hypermethylation in GSTs and biochemical recurrence. GSTM1, GSTP1 polymorphisms and hypermethylation of GSTP1, APC may be potential biomarkers for the evaluation of the probability of BCR. Further studies are warranted to validate these findings in larger cohorts with longer follow-up.

## Introduction

Prostate cancer (PCa) is the most commonly diagnosed cancer and the second leading cause of cancer-related deaths for men in the western world [1]. The unique biology of the disease poses significant challenges in the diagnosis and management of the disease. It is well recognized that widespread PSA screening has led to over-diagnosis and over-treatment of many men with indolent diseases [2], [3]. Radical prostatectomy (RP) is often performed in localized PCa. Approximately 25–40% of patients will eventually experience biochemical recurrence (BCR) after RP in a longer follow-up period [4]–[6]. PSA concentration in serum of >0.2 ng/ml on one or two occasions after a previously undetectable level after prostatectomy is regarded as BCR [7] and it is the first sign of cancer recurrence. Patients with BCR have a much worse prognosis and often develop metastasis and can die of the disease [8], [9]. So BCR have been used as an indicator of aggressive disease and immediate adjuvant treatment after RP may be beneficial for patients with high probability to develop BCR.

Several nomograms have been developed to predict subsequent risk of BCR after RP. They generally rely on known clinical and pathologic variables including PSA, Gleason score, clinical stage, and the number of positive and negative biopsy cores [4], [10], [11]. Unfortunately the collective prognostic value of these factors is unsatisfactory. Therefore, better biomarkers are urgently needed.

The glutathione-S-transferases (GSTs) are phase II enzymes involved in detoxification of reactive oxygen species and environmental carcinogens, metabolism of steroid hormones and chemotherapeutic agents [12]. Extensive research has been carried out studying the relationship between GST single nucleotide polymorphisms (SNPs) and PCa susceptibility. A meta-analysis had indicated that GST polymorphisms may predict disease susceptibility and GSTM1 null allele may be associated with the lower risk of PCa observed for Asians [13]. However, they may not be associated with disease outcome and time to recurrence [14]. As for GSTT1 polymorphism, Cotignola J, et al. [15] indicated a 2.05-fold increase of risk of BCR however the result didn’t reach a statistical significant level and studies in other institutes failed to establish such a relationship [16], [17]. Study carried out by Agalliu I, et al. [17] suggested a positive relationship between GSTM1 polymorphism and BCR while others did not comply with their findings [15], [16]. The influence of GSTP1 polymorphism on BCR has also been shown to have inconsistent findings [15]–[18] (Table 1). However, these inconsistent results may due to the limited cases included and/or the potential differences in ethnicity across these studies. For instance, study by Cotignola J, et al. [15] included only 105 patients; even for the largest studies, there are only 968 patients included [18]. So a meta-analysis of these studies is needed to yield more comprehensive understanding of GSTs polymorphisms on PCa prognosis.

**Table 1:** Characteristics of individual studies included in this meta-analysis.

No.	Author, year	Country	Ethnicity	SNP/CpG hypermethylation	Total	BCR	Non-BCR	Treatment	Sample	BCR (times of PSA>0.2)	Median follow-up (ys)	Median recurrence (ys)	Study design	Methylation test
1	Cotignola J,2012 [15]	Argentina	Caucasian	GSTM1,GSTT1,GSTP1	105	35	70	RP	Serum	1	non-BCR: 7,BCR: 3	NA	Cohort	-
2	Nock NL,2009 (1) [16]	USA	Caucasian	GSTM1,GSTT1,GSTP1	226	76	318	Mixed(RP 67%)	Serum	2	5	NA	Case-control	-
3	Nock NL,2009 (2) [16]	USA	African American	GSTM1,GSTT1,GSTP1	168				Serum	2	5	NA	Case-control	-
4	Agalliu I,2006 [17]	USA	Caucasian: 95%*	GSTM1,GSTT1,GSTP1	318	107	211	Mixed(RP 68%)	Peripheral lymphocytes	1	9.6	NA	Cohort	-
5	Dluzniewski PJ,2012 [18]	USA	Caucasian	GSTP1	968	484	484	RP	Tissue	2	4	NA	Case-control	-
6	Liu L, 2011 [22]	Canada	Caucasian	, APC	219	NA	NA	RP	Tissue	NA	NA	NA	Cohort	qmPCR
7	Ellinger J,2008 (1) [23]	Germany	Caucasian	GSTP1	122	24	98	RP	Serum	1	2.2	0.85	Cohort	reqPCR
8	Ellinger J,2008 (2) [24]	Germany	Caucasian	APC,RAR-beta	41	13	28	RP	Tissue	1	1.7	1.7	Cohort	qmPCR
9	Bastian PJ,2005 [25]	USA	Caucasian	GSTP1,, APC, RAR-beta	74	37	37	RP	Serum	1	2	3	Case-control	reqPCR
10	Rosenbaum E,2005 [27]	USA	Caucasian	GSTP1,, APC, RAR-beta	110	55	55	RP	Tissue	1	9	8	Cohort	qmPCR
11	Woodson K, 2006 [29]	USA	Caucasian 82%**	GSTP1, RAR-beta	60	11	49	RP	Tissue	2	NA	NA	Cohort	qmPCR
	Total				3037									

BCR: biochemical recurrence; Non-BCR: patients without biochemical recurrence; qmPCR: Quantitative methylation-specific PCR; reqPCR: Restriction endonuclease quantitative PCR; NA: not available.

* Caucasian 91%, African-American 9% for BCR and Caucasian 97%, African-American 3% for Non-BCR.

** Caucasian 82%, African-American 18% for BCR and Caucasian 82%, African-American 12%, Asian 6% for Non-BCR.

Epigenetic are changes in gene expression not caused by alterations in the primary sequence of the nucleotides of the gene. DNA hypermethylation is the most common epigenetic change and one of the most common molecular alterations in human cancer [19]. CpG dinucleotides can often be found in clusters called CpG islands in promoter regions. CpG islands of many genes, including tumor suppressor genes, are unmethylated in normal tissues but are methylated to varying degrees in multiple cancer types, causing silencing of gene transcription and inactivation of these tumors suppressor genes [19], [20]. Promoter regions of several genes were found to be hypermethylated in PCa using methylation-specific PCR [21]–[27]. GSTP1 promoter hypermethylation represents the best currently available DNA-based biomarker for PCa because it is present in up to 90% of prostate cancer tissues and is only rarely present in benign prostate tissue [28]. Although GSTP1 hypermethylation was reported to be predictor of early biochemical recurrence following RP, the results from different studies vary vastly. For instance, in one study hypermethylated GSTP1 in patient serum is associated with a 4.4-fold increased risk of BCR [22]. Conversely, Bastian et al. [29] and Woodson et al. [23] did not find any correlation between GSTP1 hypermethylation and BCR. However, using GSTP1 CpG island hypermethylation alone may not be able to distinguish PCa from other cancers, since GSTP1 CpG island hypermethylation has been reported in other cancers [30]. What’s more, there are evidences to believe that GSTP1 methylation could trigger “epigenetic catastrophe” [31] which involves hypermethylation of associated genes including APC (a tumor suppression gene), and RAR-beta (tumor suppressor gene involved in cell cycle and apoptosis). Also, current available studies often investigate GSTP1 CpG island hypermethylation together with these genes. So we believe it is good practice to investigate these hypermethylated genes together with GSTP1. Currently available studies reported DNA methylation levels of the promoters of GSTP1, APC and RARb2 might be associated with higher risk of BCR with inconsistent results [18], [19], [21]–[24]. Since the inconsistent results may be due to relatively small sample sizes of individual studies, we carried out a meta-analysis of the available published studies.

## Methods and Materials

### Publication search

This work was approved by the Institutional Review Board of Changhai Hospital and was performed in accordance with the PRISMA 2009 Checklist for the conduct of meta-analysis (Checklist S1). We carried out a search in Medline, Embase, and CNKI database in Chinese with ‘‘glutathione-S-transferase(GST)’’, “adenomatous polyposis coli (APC)”, “Retinoic acid receptor β (RARbeta OR RAR-beta OR RARβ)” in combination with ‘‘polymorphism OR single nucleotide polymorphisms OR SNPs’’, “methylation OR hypermethylation”, ‘‘prostate cancer OR prostate neoplasms’’ and “recurrence OR relapse OR prognosis” (last search was updated on 2012-12-12). All terms were searched as MeSH terms or key words. We checked potentially relevant publications by examining their titles and abstracts, and all studies matching the eligible criteria were retrieved. Besides the database search, the bibliographies of the selected papers and reviews were also examined manually [26].

### Criteria for inclusion and exclusion

**Figure 1:**
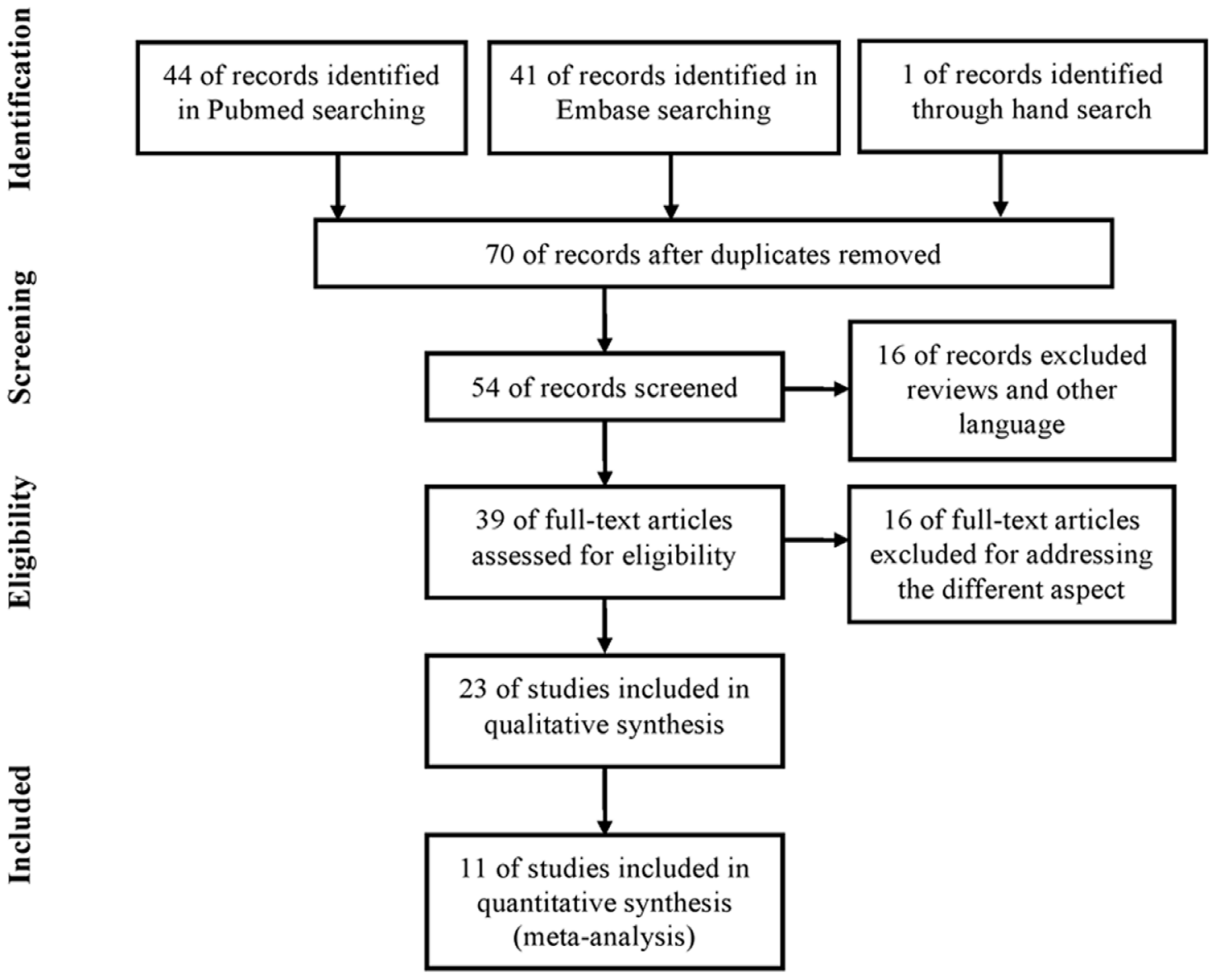
Flow chart of literature selection.

Studies included in the meta-analysis must meet all of the following criteria: (a) evaluation of the glutathione-S-transferases polymorphisms, CpG hypermethylation and prostate cancer recurrence, (b) using a cohort or case-control design, (c) using a Cox proportional hazards modeling, (d) sufficient published data for estimating an hazard ratio (HR) with 95% confidence interval (CI), and (e) article was either in English or Chinese. Accordingly, the exclusion criteria were: (a) reviews and repeated literatures, (b) not offering the source of cases and controls and other essential information, and (c) not designed as case control or cohort studies. If studies had proper design for this meta-analysis but did not have enough data, an email was sent to the authors for further supplementary data [15], [23], [24] (Figure 1). In the searching period, 44 and 41 records were included in the Pubmed and Embase. And one article was found through hand search of the citations of included articles [23]. 70 articles remains after duplication removed while 16 of them were excluded because they are review articles or written in other languages. We screened the remaining 54 articles and found that 15 of these studies focus on diagnosis of PCa and other aspect of these SNPs or hypermethylation rather than time to BCR (e.g. the proportion of more aggressive PCa or the chance of developing castration hormone refractory prostate cancer). In the remaining 23 studies for qualitative analysis, 12 articles failed to be eligible for quantitative synthesis because they didn’t provide the HRs and 95%CIs for data extraction.

### Data extraction

Data were independently abstracted by two investigators (RC and TM) using a standard protocol and data-collection form in accordance to the criteria stated above. Differences among evaluators were resolved by discussion and rereading with the third investigator (SR). The following information was extracted from each included study using a standardized data collection protocol (Table 1, Table 2): the surname of first author, year of publication, country, ethnicity, and number of cases in the cohort, number of cases with biochemical recurrence (BCR), design of the study, initial treatment, sample source, median time of follow-up, the minor allele frequency, the method used assessing methylation as well as median time to tumor recurrence. The definition of BCR in these included studies is slightly different. PSA concentration in serum of >0.2 ng/ml on one occasions after a previously undetectable level after prostatectomy is regarded as BCR in some studies while there are three studies define two consecutive PSA value of >0.2 ng/ml as BCR (Table 3). However, in clinic situation, we usually will let the patients take another PSA test in a short time to confirm previous finding so the influence between the two standards are not significant.

**Table 2:** Characteristics of patients involved in individual studies.

No.	Author, year	Age at diagnosis(ys)		PSA at diagnosis(ng/ml)		Pathologic Gleason score (%) in cases					
						BCR			Non-BCR		
		BCR	Non-BCR	BCR	Non-BCR	<7(%)	7(%)	>7(%)	<7(%)	7(%)	>7(%)
1	Cotignola J,2012 [15]	65 (49–74)		6.87 (0.77–28.90)		52(49.5)	48(47.5%)	5(4.8)	52(49.5)	48(47.5)	5(4.8)
2	Nock NL,2009 (1) [16]	60.8±6.0	61.0±6.8	10.6±9.6	6.0±4.3	NA					
3	Nock NL,2009 (2)[16]					NA					
4	Agalliu I,2006 [17]	57.5 ±4.8	57.8±4.4	NA	NA	55(38.5)	57(39.9)	29(20.3)	282(70.3)	114(25.2)	15(3.7)
5	Dluzniewski PJ,2012 [18]	58.9±6.2	59.0±5.9	12.0±9.5	10.9±8.4	72(14.9)	296(61.2)	116(24.0)	73(15.1%)	305(63.0)	106(21.9)
6	Liu L, 2011 [22]	61.4(41.5–75.9)		NA	NA	102(46.6)	98(44.7)	19(8.7)	102(46.6%)	98(44.7)	19(8.7)
7	Ellinger J,2008 (1) [23]	66(49–79)		<4: 6.6%; 4–10: 58.2%; >10:32.8%		78(63.9)	24(19.7)	20(16.4)	78(63.9%)	24(19.7)	20(16.4)
8	Ellinger J,2008 (2) [24]	66(49-79)		NA	NA	78 (63.9)	24 (19.7)	20 (16.4)	78 (63.9%)	24 (19.7)	20 (16.4)
9	Bastian PJ,2005 [25]	58.6(48–70)	59.7(43–71)	9.9(1.8–38)	7.9(1.4–23.9)	24(44)	25(46)	6(9)	34(63)	14(26)	6(11)
10	Rosenbaum E,2005 [27]	59.5(46–72)		NA	NA	0	74(100)	0	0	74(100)	0
11	Woodson K, 2006 [29]	66.5 ±3.5	64.6±6.6	5.4±3.6	8.1±12.2	2 (18.2)	9(81.8)		27 (55.1)	22 (44.9)	

BCR: biochemical recurrence; Non-BCR: patients without biochemical recurrence; NA: not available.

**Table 3:** Results of involved studies.

No.	Author, year	Gene		HR and 95%CI	P	Adjusted model	MAF
		SNP	Hypermethylation Status				
1	Cotignola J,2012 [15]	M1 null vs. present	-	0.97(0.47–2.01)	0.94	A	44.70%
2	Nock NL,2009 (1)[16]	M1 null vs. present	-	1.61(0.89–2.96)	0.11	B	42.10%(BCR),38.70%(Non-BCR)
3	Nock NL,2009 (2)[16]	M1 null vs. present	-	1.11(0.44–2.4)	0.95	B	42.10%(BCR),38.70%(Non-BCR)
4	Agalliu I,2006 [17]	M1 null vs. present	-	1.32(0.89–1.96)	NA	C	51.89%
1	Cotignola J,2012 [15]	T1 null vs. present	-	2.05(0.92–4.54)	0.08	A	21.20%
2	Nock NL,2009 (1)[16]	T1 null vs. present	-	0.55(0.21–1.40)	0.2	B	19.70%(BCR),21.10%(Non-BCR)
3	Nock NL,2009 (2)[16]	T1 null vs. present	-	2.3(1.01–5.18)	0.04	B	19.70%(BCR),21.10%(Non-BCR)
4	Agalliu I,2006 [17]	T1 null vs. present	-	1.09 (0.68–1.77)	NA	D	16.67%
1	Cotignola J,2012 [15]	GSTP1 AG vs. AA	-	0.85 (0.33–2.17)	0.73	A	39.80%
2	Nock NL,2009 (1)[16]	GSTP1 AG vs. AA	-	0.54(0.27–1.08)	0.08	B	44.70%(BCR),50.00%(Non-BCR)
3	Nock NL,2009 (1)[16]	GSTP1 AG vs. AA	-	1.71(0.64–4.55)	0.28	B	44.70%(BCR),50.00%(Non-BCR)
4	Agalliu I,2006 [17]	GSTP1 AG vs. AA	-	1.01(0.66–1.53)	NA	C	44.03%
5	Dluzniewski PJ,2012 [18]	GSTP1 AG vs. AA	-	1.40(1.06–1.86)	NA	Univariate	39.58%
1	Cotignola J,2012 [15]	GSTP1 GG vs. AA	-	2.73 (0.89–8.38)	0.08	A	10.70%
2	Nock NL,2009 (1)[16]	GSTP1 GG vs. AA	-	0.96(0.40–2.28)	0.93	B	21.10%(BCR),13.20%(Non-BCR)
3	Nock NL,2009 (2)[16]	GSTP1 GG vs. AA	-	2.1(0.66–6.67)	0.21	C	21.10%(BCR),13.20%(Non-BCR)
4	Agalliu I,2006 [17]	GSTP1 GG vs. AA	-	0.91(0.49–1.66)	NA	D	10.38%
5	Dluzniewski PJ,2012 [18]	GSTP1 GG vs. AA	-	1.35(0.84–2.16)	NA	Univariate	11.73%
7	Ellinger J,2008 (1) [23]	-	GSTP1 hypermethylation	1.25(0.58–2.69)	0.58	Univariate	-
8	Ellinger J,2008 (2) [24]	-	GSTP1 hypermethylation	1.02(0.96–1.08)	0.5	Univariate	-
9	Bastian PJ,2005 [25]	-	GSTP1 hypermethylation	0.34(0.13–0.88)	0.03	Margin, age, lymph node status	-
10	Rosenbaum E,2005 [27]	-	GSTP1 hypermethylation	0.34(0.13–0.88)	0.03	Univariate	-
11	Woodson K, 2006 [29]	-	GSTP1 hypermethylation	5.31 (0.63–45.07)	0.13	Univariate	-
6	Liu L, 2011 [22]	-	APC hypermethylation	2.22(0.78–6.32)	0.14	Margin, age, Gleason score,tumor stage	-
8	Ellinger J,2008 (2) [24]	-	APC hypermethylation	1.47(0.03–72.67)	0.85	Univariate	-
10	Rosenbaum E,2005 [27]	-	APC hypermethylation	1.60(0.80–3.19)	0.18	Univariate	-
9	Ellinger J,2008 (2) [24]	-	RAR(beta) hypermethylation	1.00(1.00–1.00)	0.34	Univariate	-
10	Rosenbaum E,2005 [27]	-	RAR(beta) hypermethylation	1.22(0.59–2.52)	0.59	Univariate	-
11	Woodson K, 2006 [29]	-	RAR(beta) hypermethylation	3.34(0.66–17.29)	0.14	Univariate	-

A: Adjusted for age, margin, Gleason score, tumor stage, PSA, family history of PCa, smoking status.

B: Adjusted for age, tumor stage, tumour grade, PSA, smoking satus.

C: Adjusted for age, Gleason score, tumor stage, PSA,familiy history of PCa, smoking status, and RP status.

MAF: minor allele frequency; BCR: biochemical recurrence; Non-BCR: patients without biochemical recurrence; NA: not available.

There are two groups of patients in study by Nock et al. The data set of Nock NL, 2009 (1) is consist of Caucasian population and Nock NL, 2009 (2) data set is consist of African-Americans.

Most studies on GSTs polymorphisms uses multivariable Cox proportional hazards model. So we extract HR and 95%CI in the multivariable model that were adjusted for age, Gleason score, tumor stage at diagnosis, PSA screening history, smoking, and radical prostatectomy status as the multivariable model listed in Table 3. On the other hand, most studies on hypermethylation and PCa recurrence uses the univariate analysis and so that we extract HR and 95%CI in the univariate analysis or the closest to it (Table 3). So the analysis of these SNPs is mainly based on multivariable model and the analysis of hypermethylation changes is mainly based on univariable model. The ethnicity was categorized as Caucasian, African-American or mixed population. The percentage of each population in the mixed population has been specified in Table 1. In cases of publications by the same author, inquiries were sent to the author to clarify if there were overlaps of patients.

### Statistical Analysis

The strength of the association between these polymorphisms or hypermethylation of promoter region and time to PCa biochemical recurrence was measured by HRs with 95% CIs. Odds ratios (ORs) or relative risks (RRs) measure only the number of events and take no account of when they occur are appropriate for measuring dichotomous outcomes, but less appropriate for analyzing time-to-event outcomes. The statistical significance of the summarized HR was determined by the Z-test. For GSTM1 and GSTT1 null polymorphisms, we estimated the impact of the ‘‘Null’’ genotype on time to recurrence, compared with the ‘‘Present’’ genotype. When it comes to GSTP1, the risk of biochemical recurrence evaluated is GSTP1 “AG vs. AA” and “GG vs. AA”, respectively. We didn’t evaluate other models (e.g. GG and AG vs. AA) because the data from included studies provides not sufficient data. As for hypermethylation of GSTP1 and other genes included, the risk of “promoter hypermethylation” compared with “not hypermethylation” was estimated. Homogeneity was evaluated by x2-based Q-test. If this test is rejected using a p-value cut point of 0.10 or less then there is sufficient evidence for the existence of heterogeneity and a lack of homogeneity. In this situation we utilize a random-effects model (the DerSimonian and Laird method) [32] which takes into account the between study variation. If p>0.1, it indicate homogeneity among these studies. There is a necessity to conduct the fixed-effect model (the Mantel-Haenszel method) [33] and we would also report the results of the random effects models as a form of sensitivity analysis to ensure that they are not substantially different. Sensitivity analysis was performed to assess the stability of the results. Begg’s funnel plot and Egger’s test were performed to assess the publication bias of literatures [34]; P<0.05 was considered statistically significant. All statistical tests for this meta-analysis were performed with STATA (version 11.0; Stata Corporation, College Station, TX).

## Results

### Study characteristics

All potentially eligible studies investigating the relationship between GST polymorphisms or the methylation of promoter region and prostate cancer recurrence were identified. During the extraction of data, 12 articles were excluded, because they did not provide essential data leaving 11 eligible articles including 12 data sets involving 3,037 prostate cancer patients [15]–[18], [22]–[27] (Figure 1, Table 1). For GSTM1 and GSTT1 null polymorphisms, 817 patients in four groups were included from three studies [15]–[17] and for GSTP1 polymorphism, 1785 patients were included from five studies [15]–[18]. For GSTP1 heypermethylation five studies with 347 patients were included. For APC and RAR(beta), there are three studies included involving 293 and 144 patients, respectively. Several studies included patients of Caucasian descent while other studies included mixed races [17], [26] and African-American population [16]. The ethnicity of the studies in mixed population is mainly Caucasian (95% and 82% respectively). Most articles studying polymorphisms used blood samples for genotyping assay except for one, which used tissue [18]. On the other hand, all studies on hypermethylation of gene promoter are genotyped with prostate cancer tissue except one, which used serum [25].

### Test of homogeneity

There was significant heterogeneity across the studies of the GSTT1 null polymorphism, GSTP1 AG vs. AA polymorphism and GSTP1 hypermethylation. So a random-effect model was utilized to analyze these data and the source of heterogeneity was further explored in the sensitivity test. For other meta-analysis, the fixed-effect model was implied and the results of random-effect model have been compared as a type of sensitivity test.

### Quantitative synthesis

**Figure 2:**
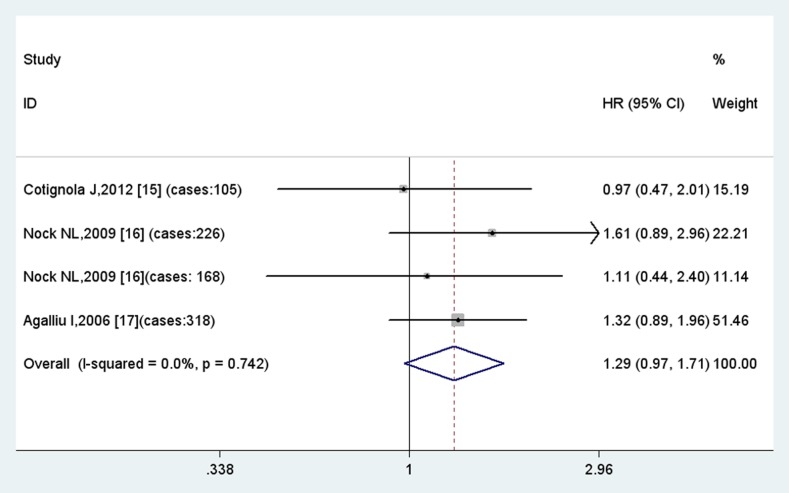
Results of meta-analysis of GSTM1 null polymorphism.

For GSTM1 null polymorphism, none of the four included studies suggested a significant association with biochemical recurrence of prostate cancer. However, the meta-analysis in the fix model indicates that this polymorphism is associated with a 1.3-fold risk for biochemical recurrence with borderline significance (HR = 1.29, 95%CI = 0.97–1.71, p = 0.08) (Figure 2). So we may hypothesize that in a larger population GSTM1 null polymorphism may act as slight hazard for prostate cancer BCR.

**Figure 3:**
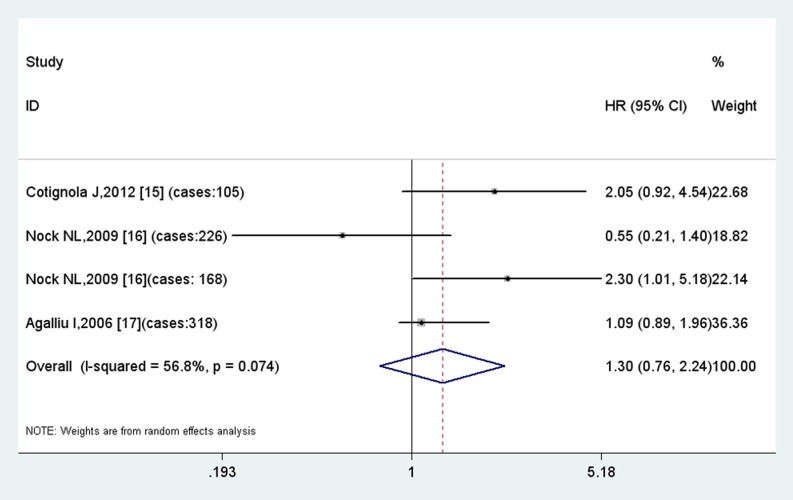
Results of meta-analysis of GSTT1 null polymorphism.

The meta-analysis of GSTT1 null polymorphism did not show significant association among 4 studies with relatively large heterogeneity (P_h_ = 0.08, I^2^ = 0.57). The results indicate GSTT1 null polymorphism to be a modest risk factor for biochemical recurrence (Figure 3).

**Figure 4:**
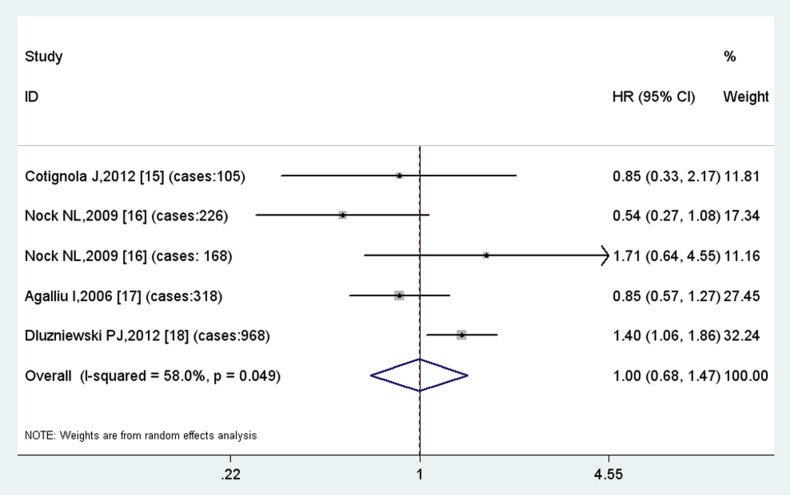
Results of meta-analysis of GSTP1 AG vs. AA polymorphism.

**Figure 5:**
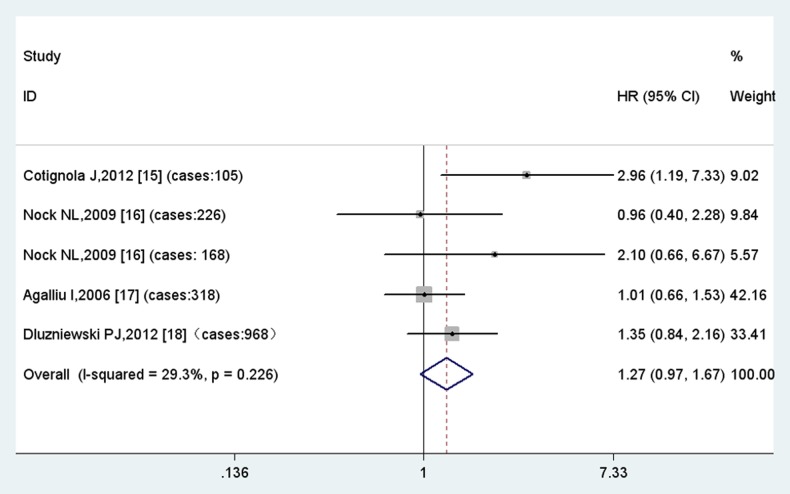
Results of meta-analysis of GSTP1 GG vs. AA polymorphism.

The overall HR with its 95% CI showed no statistically significant association between the GSTP1 AG vs. AA polymorphism and time to biochemical recurrence using a random effect model (HR =  1.00, 95% CI  =  0.68–1.47) (Figure 4). In the subgroup analysis by ethnicity, no statistically significant association was found among Caucasians either. On the contrary, GG vs. AA polymorphism is correlated with the risk of recurrence with borderline significance (HR = 1.27, 95%CI =  0.97–1.67, p = 0.09), indicating a modest risk for patients of GSTP GG polymorphism to have a recurrence (Figure 5).

**Figure 6:**
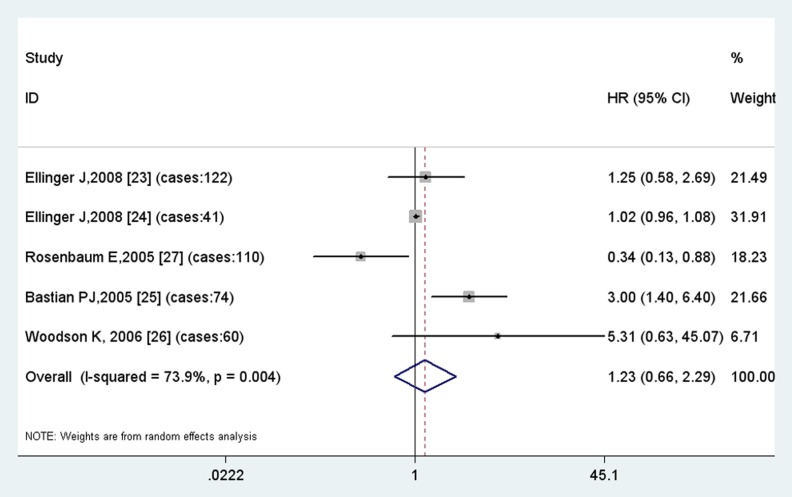
Results of meta-analysis of APC promoter hypermethylation.

**Figure 7:**
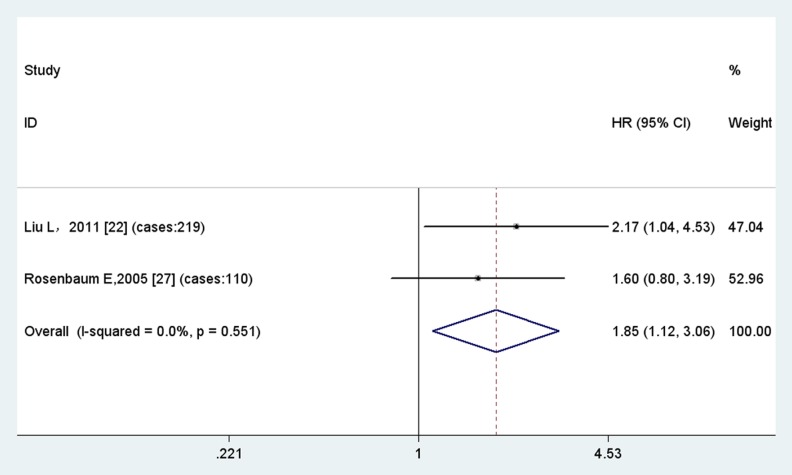
Results of meta-analysis of GSTP1 hypermethylation.

**Figure 8:**
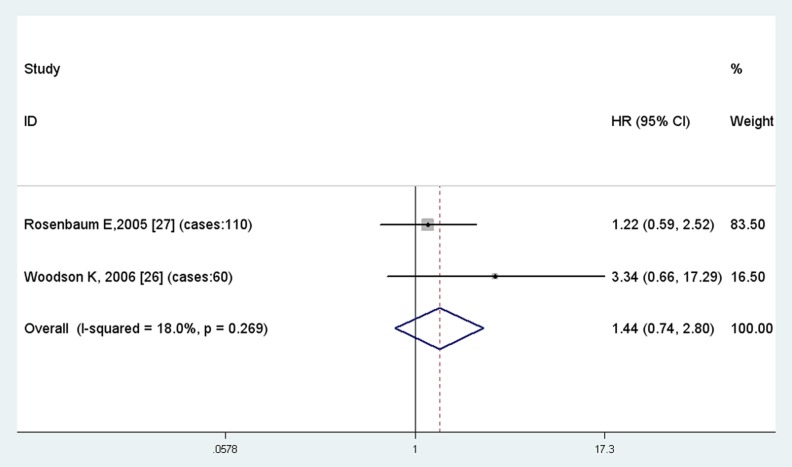
Results of meta-analysis of RAR-beta hypermethylation.

APC hypermethylation was associated with an increase risk of prostate cancer biochemical recurrence (HR =  1.23, 95% CI  =  1.07–1.42) (Figure 6, Table 4). Nevertheless, results showed no significant association between GSTP1 and RAR-beta promoter region hypermethylation and the recurrence after RP (Figure 7, Figure 8). GSTP1 and RAR-beta hypermethylation appears to be associated with a higher risk of biochemical recurrence (HR = 1.23 and 1.44 respectively) (Table 4).

**Table 4:** Main Results of meta-analysis.

No.	Gene	SNP/Epigenetic	No. of studies	No. of patients	Model	Test of association		Test of heterogeneity		
						HR (95% CI)	P-value	Q	P-value	I^2^
1	GSTM1	Null vs. present	4	817	Fixed	1.29 (0.97, 1.71)*	0.08	9.53	0.74	0
2	GSTT1	Null vs. present	4	817	Random	1.31(0.76, 2.24)	0.33	6.95	0.07	0.57
3	GSTP1	AG vs. AA	5	1785	Random	1.00 (0.68,1.47)	0.99	9.53	0.05	0.58
4	GSTP1	GG vs. AA	5	1785	Fixed	1.27(0.97,1.67)*	0.09	5.66	0.23	0.29
5	GSTP1	Hypermethylation vs. Non-hypermethylation	5	347	Random	1.23(0.66,2.29)	0.52	15.34	0.01	0.74
6	APC	Hypermethylation vs. Non-hypermethylation	3	293	Fixed	1.85(1.12,3.06)*	0.02	0.36	0.55	0
7	RAR(beta)	Hypermethylation vs. Non-hypermethylation	3	144	Fixed	1.44(0.74,2.80)*	0.28	1.22	0.27	0.18

* HRs and 95%CIs in the random-effect model are: 1.29(0.97, 1.71), 1.33(0.94, 1.9), 1.85(1.12, 3.06), and 1.53(0.67, 3.49) for GSTM1, GSTP1 GG vs. AA,, APC and RAR(beta), respectively.

### Publication bias

Begg’s funnel plot and Egger’s test were performed to assess the publication bias of the currently available literature. The shapes of the funnel plot for the comparison of all the gene polymorphisms and promoter hypermethylations appeared symmetrical. Egger’s test was used to provide statistical evidence for funnel plot symmetry. The p-values of the Egger’s tests are 0.55 for GSTM1, 0.78 for SGTT1, 0.47 for GSTP1 AG vs. AA and 0.60 for GSTP1 GG vs. AA The results did not suggest any evidence of publication bias (Figure S1).

### Sensitivity analysis

For all gene variants, sensitivity analysis was performed by excluding one or more studies at one time. We estimated the summarized effect in stratified analysis by race, sample type or method to test methylation. Results of stratified analysis have been listed in Table 5.

**Table 5:** Results of sensitivity analysis.

No.	Gene	SNP/Epigenetic	Subgroup	Model	Test of association		Test of heterogeneity		
					HR (95% CI)	P-value	Q	P-value	I^2^
1	GSTM1	Null vs. present	Sanmple: serum	Fixed	1.26(0.84,1.89)	0.26	1.22	0.54	0.00
2	GSTM1	Null vs. present	Ethnicity: Caucasian	Fixed	1.31(0.98,1.78)	0.07	1.11	0.57	0.00
3	GSTM1	Null vs. present	Treatment:mixed(RP:67–68%)	Fixed	1.02(0.59,1.78)	0.93	0.06	0.81	0.00
4	GSTT1	Null vs. present	Sanmple: serum	Random	1.42(0.61,3.32)	0.42	5.94	0.05	0.66
5	GSTT1	Null vs. present	Ethnicity: Caucasian	Fixed	1.12(0.80,1.56)	0.51	4.38	0.11	0.54
6	GSTT1	Null vs. present	Treatment:mixed(RP:67–68%)	Fixed	1.14(0.60,2.19)	0.69	5.15	0.08	0.61
7	GSTP1	AG vs. AA	Sanmple: serum	Random	1.04(0.62,1.76)	0.87	7.25	0.06	0.59
8	GSTP1	AG vs. AA	Ethnicity: Caucasian	Random	0.93(0.60,1.43)	0.73	8.73	0.03	0.66
9	GSTP1	GG vs. AA	Sanmple: serum	Fixed	1.50(1.05,2.15)	0.03	3.67	0.30	0.18
10	GSTP1	GG vs. AA	Ethnicity: Caucasian	Fixed	1.23(0.93,1.63)	0.14	4.89	0.18	0.39
11	GSTP1	Hypermethylation vs. Non-hypermethylation	Sample: tissue	Random	1.02(0.29,3.59)	0.98	7.30	0.03	0.73
12	GSTP1	Hypermethylation vs. Non-hypermethylation	Sample: serum	Fixed	1.94(1.13,3.34)	0.02	2.54	0.11	0.61
13	GSTP1	Hypermethylation vs. Non-hypermethylation	Method: qmPCR	Random	0.90(0.33,2.48)	0.84	7.36	0.03	0.73
14	GSTP1	Hypermethylation vs. Non-hypermethylation	Method: reqPCR	Fixed	1.94(1.13,3.34)	0.02	2.54	0.11	0.61

For GSTM1 null polymorphism, when we perform stratified analysis in studies in Caucasian, the summarized results indicated a moderate hazard for GSTM1 null polymorphism at a borderline significance (HR = 1.31, 95%CI = 0.98–1.78, p = 0.07). As for GSTT1 null polymorphism, results of any stratification failed to yield a significant association with prostate cancer BCR. GSTP1 GG polymorphism showed a 1.5-fold risk of BCR over GSTP1 AA when the analysis is confined to studies using serum sample (HR = 1.50, 95%CI = 1.05–2.15, p = 0.03). Subgroup analysis of studies in Caucasians has achieved similar results with a lower HR and a borderline significance similar with results of the overall analysis (p = 0.14 and p = 0.09, respectively). GSTP1 hypermethylation status was not significant associated with BCR in the overall analysis, however, when we confine the analysis to those carried out using serum samples with restriction endonuclease quantitative PCR to test methylation level, hypermethylation act as a significant risk factor for BCR (HR = 1.94, 95%CI = 1.13–3.34, p = 0.02). When we use the random-effect model for those studies with a low heterogeneity, the results are quite close to the data we got from the fixed-effect model (Table 4). Thus we may have more evidence to believe it is appropriate to imply the fixed-effect model.

## Discussion

In current standard of care, biochemical recurrence after RP serves as a trigger point for further treatment; therefore any biomarker that is correlated with biochemical recurrence would be a valuable tool for the clinical management of the disease. Polymorphism of GSTs has been extensively studied unveiling a possible association with prostate cancer susceptibility and risk of biochemical recurrence. This meta-analysis supports the association with GSTM1 and GSTP1 polymorphism with an increased risk of BCR with borderline significance. The proteins GSTM1 and GSTP1 encode are known to have an important impact in modification of some enzymes. These enzymes may have function in the detoxification of electrophilic compounds, including carcinogens, therapeutic drugs, environmental toxins and products of oxidative stress, by conjugation with glutathione [35]. The polymorphisms of GSTM1 and GSTP1 may influence the function of these enzymes in carcinogenicity of prostate. Additionally, hypermethylation status has been used as effective biomarkers in some pioneering studies with satisfactory results [36]. In one study included in the meta-analysis, quantitative methylation assessment of a multiplex panel of markers, consisting of APC, HOXD3 and TGFβ2, outperforms any single currently available biomarker [22]. In another study, APC exhibits very high NPV (negative predictive value) in men with initial negative biopsy but high suspicion for cancer suggesting that methylation markers have the potential to eliminate up to 30% of re-biopsies after an initial negative biopsy [36].

We put special emphasis on GSTP1, because its methylation has been shown to occur early in high-grade prostatic intraepithelial neoplasia (HGPIN), suggesting the possibility of using GSTP1 to detect very early stage of recurrence [37]. Moreover, some researchers have hypothesized that GSTP1 methylation could trigger “epigenetic catastrophe” [31] which involves hypermethylation of additional genes including APC, and RAR-beta. Further investigations should target more genes including EPB41L3, HOXD3, CD44, PTGS2 and other genes that may be involved in this hypermethylation process [37].

In this meta-analysis, we suggest APC hypermethylation can pose a modest hazard for BCR after RP. The results also indicated that GSTP1 hypermethylation tested in serum may be an effective indicator for BCR after RP. GSTP1 GG polymorphism tested in serum has been illustrated to poses a hazard to BCR in overall population with a borderline significance and significant results were yield in the studies using serum as test sample. Taking the limited sample size of included studies, we may believe that if more cases are enrolled this effect may be more significant.

In the 11 studies included, 4 are designed as case-control studies and 7 are cohort studies. All the case-control studies have selected the proper controls and the cohort studies are also well-designed. Furthermore, nine studies indicated a median time follow-up ranging from 1.7 to 9 years and two studies failed to offer the median time follow-up. However, the median time to BCR after RP ranged from 1.7 to 8 years and in 7 studies this data was not reported, suggesting the possibility of insufficient follow-up. In the publication search period we excluded articles in languages other than English and Chinese. When considering studies of other language, only one study in German was qualified; however, there was inadequate data in the abstract to be included.

In interpreting the results, some cautions should be applied. First, the heterogeneity and small sample size may have distorted this meta-analysis. For instance, a few published studies lacked the essential data required and not all the articles set RP as an initial treatment, making the effects of different treatments on the time to BCR and rate of BCR unclear. Further studies should discriminate between various treatments and focus on the BCR after a single therapeutic modality such as RP. Similarly, there is some heterogeneity in the aspect of ethnicity, as some of the studies investigated mixed population. Secondly, currently available studies failed to investigate the relationship between race and gene polymorphisms and methylation. Subsequent studies should concentrate on exploring the genetic and epigenetic differences that exist among the different races. Thirdly, although available genetic data suggest an increased risk for BCR with APC, GSTM1 and GSTP1 promoter methylation, we still lack the knowledge of their gene-environment interactions. Further studies are warranted to confirm these findings.

## Conclusion

In conclusion, to our knowledge this is the first meta-analysis evaluating the polymorphisms and methylation in GSTs and biochemical recurrence. We confirmed that APC CpG hypermethylation poses a modest hazard for BCR after RP. We also suggest GSTP1 polymorphism and CpG hypermethylation tested in serum are probably associated with BCR. There are the potential implications of these SNPs and epigenetic change for evaluation of the probability of BCR. Further studies are warranted to validate these findings in a larger cohort with a longer follow-up.

## Supporting Information

Figure S1 **Funnel plot of publication bias.**
(DOCX)

Checklist S1 **PRISMA 2009 Checklist.**
(DOC)
