# Supplementary figures and images for: Impact of Glutathione-S-Transferases (GST) Polymorphisms and Hypermethylation of Relevant Genes on Risk of Prostate Cancer Biochemical Recurrence: A Meta-Analysis
Source: PLoS One. 2013 Sep 23;8(9):e74775. doi: 10.1371/journal.pone.0074775 (PMC3781159; doi:10.1371/journal.pone.0074775)

APC:


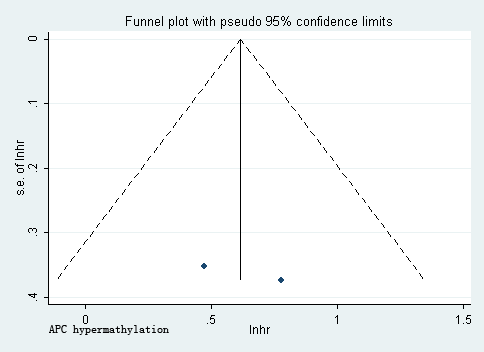


GSTM1:


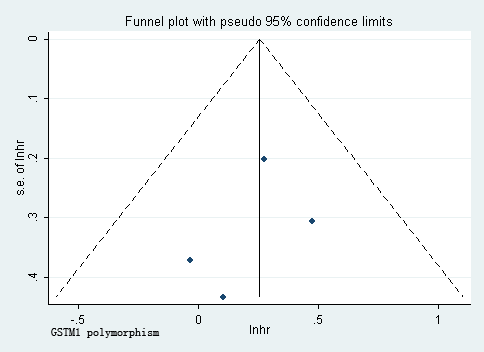


GSTP1 GA vs AA


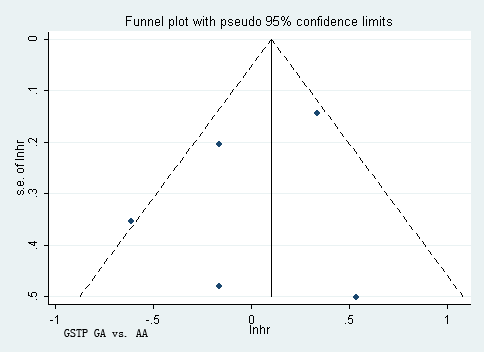


GSTP1 GG vs AA:


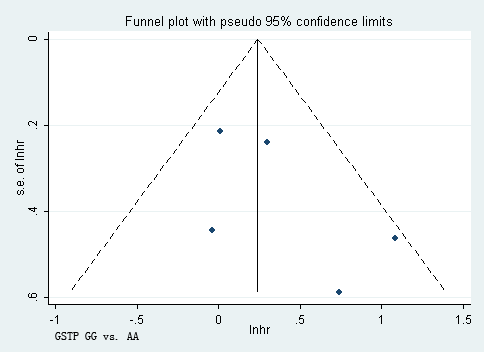


GSTT1:


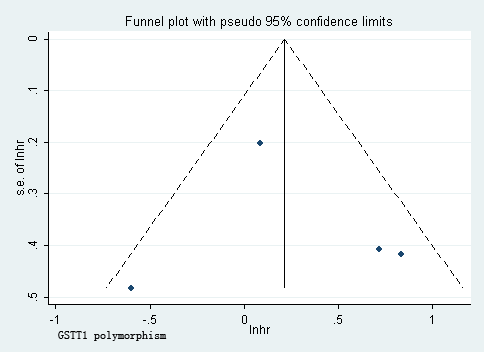


GSTP1:


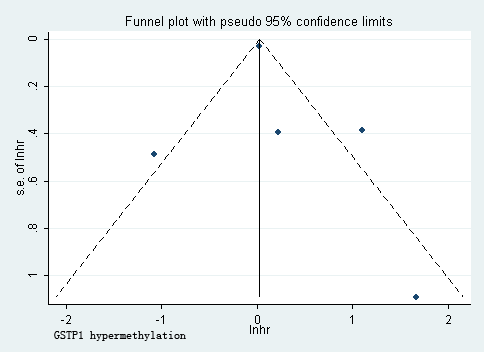


RAR-beta:
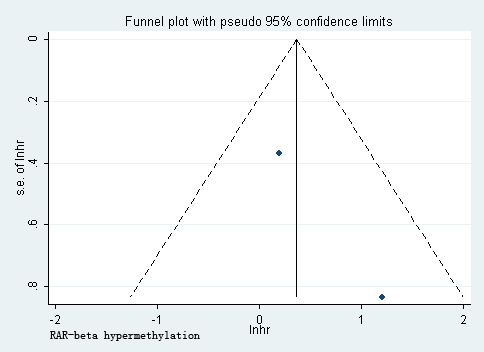


RASSF1A:


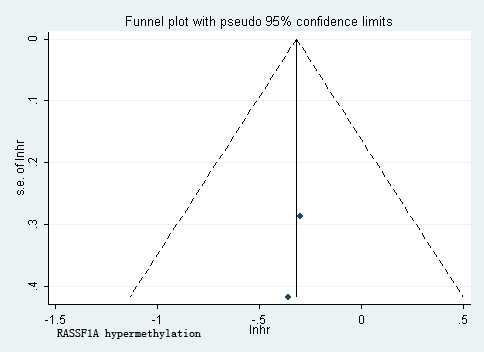

Supplement: Figure S1 — Funnel plot of publication bias. (DOCX) [file pone.0074775.s001.docx]
