# Supplementary material for: Impact of Glutathione-S-Transferases (GST) Polymorphisms and Hypermethylation of Relevant Genes on Risk of Prostate Cancer Biochemical Recurrence: A Meta-Analysis
Source: PLoS One. 2013 Sep 23;8(9):e74775. doi: 10.1371/journal.pone.0074775 (PMC3781159; doi:10.1371/journal.pone.0074775)
Supplement: Checklist S1 — PRISMA 2009 Checklist. (DOC) [file pone.0074775.s002.doc]

| **Section/topic** | **#** | **Checklist item** | **Reported on page #** |
| --- | --- | --- | --- |
| **TITLE** | | |  |
| Title | 1 | Impact of glutathione-S-transferases (GST) polymorphisms and hypermethylation of relevant genes on risk of prostate cancer biochemical recurrence: a meta-analysis | 1 |
| **ABSTRACT** | | |  |
| Structured summary | 2 | Introduction: Accurate prediction of the biochemical recurrence (BCR) is critical for patients after definitive therapy for prostate cancer. Glutathione-S-transferases polymorphisms and hypermethylation of certain genes were reported to be associated with BCR but the reported results are inconsistent. To evaluate the relationship of glutathione-S-transferases polymorphisms and hypermethylation of relevant genes on the risk of prostate cancer biochemical recurrence, we carried out a meta-analysis of the published studies.  Methods and materials: We performed a search in Medline, Embase and CNKI database with ‘‘glutathione-S-transferase (GST)’’, “adenomatous polyposis coli (APC)”, “Ras association domain family protein 1 isoform A (RASSF1A)”, “Retinoic acid receptorβ (RARbeta/RAR-beta/RARβ)” in combination with ‘‘polymorphism OR single nucleotide polymorphisms OR SNPs’’, “methylation OR hypermethylation” , ‘‘prostate cancer OR prostate neoplasms’’ and “recurrence OR relapse OR prognosis”. Languages were restricted to English and Chinese.  Results: Our study included 4 case-control studies and 7 cohort studies including 12 data sets and 2,961 prostate cancer patients. We confirmed that APC hypermethylation is associated with a modest hazard for biochemical recurrence after radical prostatectomy (RP) (HR= 1.23, 95% CI = 1.07–1.42). We also identified a possible correlation between GSTP1 GG vs. AA polymorphism and GSTM1 null polymorphism and prostate cancer biochemical recurrence risk with borderline significance.  Conclusion: To our knowledge, this is the first meta-analysis establishing a potential association between biochemical recurrence and gene polymorphisms and promoter hypermethylation. GSTM1, GSTP1 polymorphisms and hypermethylation of relevant genes may be potential biomarkers for the evaluation of the probability of BCR. Further studies are warranted to validate these findings in larger cohorts with longer follow-up. | 2 |
| **INTRODUCTION** | | |  |
| Rationale | 3 | Glutathione-S-transferases polymorphisms and hypermethylation of certain genes were reported to be associated with BCR but the reported results are inconsistent. | 3 |
| Objectives | 4 | To evaluate the relationship of glutathione-S-transferases polymorphisms and hypermethylation of relevant genes on the risk of prostate cancer biochemical recurrence, we carried out a meta-analysis of the published studies. | 3 |
| **METHODS** | | |  |
| Protocol and registration | 5 | No protocol and registration. | NA |
| Eligibility criteria | 6 | Studies included in the meta-analysis had to meet all the following criteria: (a) evaluation of the glutathione-S-transferases polymorphisms, and prostate cancer recurrence, (b) using a cohort or case-control design, (c) using a Cox proportional hazards modeling, (d) sufficient published data for estimating an hazard ratio (HR) with 95% confidence interval (CI), and (e) article was either in English or Chinese. Accordingly, the exclusion criteria were: (a) reviews and repeated literatures, (b) not offering the source of cases and controls and other essential information, and (c) not designed as case control or cohort studies. | 5 |
| Information sources | 7 | We carried out a search in Medline, Embase, and CNKI database in Chinese (last search was updated on 2012-12-12). | 4 |
| Search | 8 | ‘‘glutathione-S-transferase(GST)’’, “adenomatous polyposis coli (APC)”, “Ras association domain family protein 1 isoform A (RASSF1A)”, “Retinoic acid receptor β (RARbeta/RAR-beta/RARβ)” in combination with ‘‘polymorphism OR single nucleotide polymorphisms OR SNPs’’, “methylation OR hypermethylation” , ‘‘prostate cancer OR prostate neoplasms’’ and “recurrence OR relapse OR prognosis” | 4 |
| Study selection | 9 | Two investigators (RC and SR) screened each of the titles, abstracts, and full texts to determine inclusion independently. The results were compared and disagreements were resolved by consensus. | 5 |
| Data collection process | 10 | Data were independently abstracted by two investigators (RC and TM) using a standard protocol and data-collection form in accordance to the criteria stated above. | 6 |
| Data items | 11 | The following information was extracted from each included study using a standardized data collection protocol: the surname of first author, year of publication, country, ethnicity, and number of cases in the cohort, number of cases with biochemical recurrence (BCR), design of the study, initial treatment, sample source, median time of follow-up, as well as median time to tumor recurrence. | 5 |
| Risk of bias in individual studies | 12 | The quality of included studies was evaluated. | 11 |
| Summary measures | 13 | The principal summary measures are odds ratios (HRs) and 95% confidence intervals (CIs). | 6 |
| Synthesis of results | 14 | The strength of the association between these polymorphisms or hypermethylation of promoter region and time to PCa biochemical recurrence was measured by HRs with 95% CIs. | 6 |

Page 1 of 2

| **Section/topic** | **#** | **Checklist item** | **Reported on page #** |
| --- | --- | --- | --- |
| Risk of bias across studies | 15 | An estimate of potential publication bias was carried out by Begg’s funnel plot and Egger’s regression test (p < 0.05 was considered representative of statistically significant publication bias). | 6 |
| Additional analyses | 16 | Sensitivity analysis was carried out for each polymorphism or methylation changes. When a higher heterogeneity, a subgroup analysis was performed. | 6 |
| **RESULTS** | | |  |
| Study selection | 17 | 23 publications were included in the qualitative analysis according to the inclusion criteria. During the extraction of data, 12 articles were excluded, because they did not provide data, the contents lacked a control, or the contents were mainly associated with diagnosis, leaving 11 eligible articles including 12 data sets involving 2,961 prostate cancer patients (Flow chart Figure 1) | 7 |
| Study characteristics | 18 | Table 1 shows the studies included in the meta-analysis and their main characteristics. | NA |
| Risk of bias within studies | 19 | The quality of included studies was evaluated. There are 9 studies that indicated a median time follow-up ranging from 1.7 to 9 years and two studies failed to offer the median time follow-up. However, the median time to recurrence after RP ranged from 1.7 to 8 years and in 7 studies this data was not reported, suggesting the possibility of insufficient follow-up. | 11 |
| Results of individual studies | 20 | The main results of individual studies were shown in Figure 2 and Table 2 and Table 3, respectively. | 7 |
| Synthesis of results | 21 | All results of synthesis of data were displayed in Quantitative synthesis section. | 7 |
| Risk of bias across studies | 22 | The shapes of the funnel plot for the comparison of all the gene polymorphisms and promoter hypermethylations appeared symmetrical. Egger’s test was used to provide statistical evidence for funnel plot symmetry. The results did not suggest any evidence of publication bias (Figure S2). |  |
| Additional analysis | 23 | For all gene variants, sensitivity analysis was performed by excluding one study at one time. As for GSTP1 GG vs. AA, one study took the largest weight in the analysis with a result indicating no significant differences. After exclusion of this study, the outcome changed greatly resulting in a significant association (HR= 1.502, 95%CI =1.049-2.151, p = 0.026). As discussed above, when the possible main source of heterogeneity in the GSTT1 null meta-analysis was removed, the result changed and indicated a protective effect of GSTT1 null with borderline significance (Figure 3A). For other genes, sensitivity test didn’t change the existing results. | 9 |
| **DISCUSSION** | | |  |
| Summary of evidence | 24 | GSTM1, GSTP1 polymorphisms and hypermethylation of relevant genes may be potential biomarkers for the evaluation of the probability of BCR. Further studies are warranted to validate these findings in larger cohorts with longer follow-up. | 10 |
| Limitations | 25 | First, the heterogeneity and small sample size may have distorted this meta-analysis. Further studies should discriminate between various treatments and focus on the recurrence after a single therapeutic modality. Secondly, currently available studies failed to investigate the relationship between race and gene polymorphisms and methylation. Thirdly, although available genetic data suggest an increased risk for recurrence with APC, GSTM1 and GSTP1 promoter methylation, we still lack the knowledge of their gene-environment interactions. Further studies are warranted to confirm these findings. | 11 |
| Conclusions | 26 | We confirmed that APC hypermethylation can pose a modest hazard for BCR after RP. We also suggest the potential implications of these SNPs and epigenetic changes as biomarkers for evaluation of the probability of BCR. Further studies are warranted to validate these findings in a larger cohort with a longer follow-up. | 11 |
| **FUNDING** | | |  |
| Funding | 27 | No funding was received in this research. | 12 |

*From:*  Moher D, Liberati A, Tetzlaff J, Altman DG, The PRISMA Group (2009). Preferred Reporting Items for Systematic Reviews and Meta-Analyses: The PRISMA Statement. PLoS Med 6(6): e1000097. doi:10.1371/journal.pmed1000097

For more information, visit: **www.prisma-statement.org**.

Page 2 of 2
